# Secular changes in the anthropometric and motor characteristics of Polish male university students between 2000 and 2018

**DOI:** 10.1002/ajhb.23520

**Published:** 2020-10-14

**Authors:** Robert Podstawski, Piotr Żurek

**Affiliations:** ^1^ Department of Tourism, Recreation and Ecology University of Warmia and Mazury in Olsztyn Olsztyn Poland; ^2^ Department of Physical Education and Sport in Gorzów Poznań University of Physical Education Poznań Poland

## Abstract

**Objectives:**

To determine changes in the anthropometric and motor characteristics of young males during the first 20 years of the 21st century in Poland.

**Materials and Methods:**

The study was conducted in 2000–2018 on 2691 randomly selected male university students aged 19÷25 (20.0 ± 1.1 years). The participants' body mass and height were measured, and the students participated in 13 motor ability tests. The analyzed traits were evaluated by testing the fit of linear and curvilinear functions to empirical data.

**Results:**

The students evaluated in 2018 were 1.7 cm taller than their peers tested in 2000. Body mass and BMI values continued to decrease between 2000 and 2006 (by 0.46 kg and 0.15 kg/m^2^ per year on average), whereas a steady and significant increase in both parameters was observed between 2006 and 2018 (by 0.45 kg and 0.12 kg/m^2^ per year on average). The results of motor tests were strongly correlated with body mass and BMI, and they continued to improve until 2006, after which a steady decline was observed up to 2018 when the students scored lowest in motor tests.

**Conclusions:**

The trend of increasing body height has been maintained in the studied population, but unlike body mass and BMI, the rate of increase in body height was lower than in the preceding years. A decrease in body mass and BMI is correlated with an improvement in motor tests, whereas an increase in the above parameters leads to a significant decline in all evaluated motor abilities.

## 1 INTRODUCTION

A secular trend generally refers to changes in body size, other anthropometric characteristics and motor fitness over one or several decades (Claessens & Lefevre, 1992). Most studies of secular trends have examined stature and body mass, whereas changes in motor fitness levels were less frequently investigated. Children and adolescents evaluated between the 1960s and the 1980s were taller, heavier and achieved higher levels of motor fitness in several tests (mainly handgrip strength, running and jumping) than the subjects who had been studied in the first half of the 20th century (Malina, 1984; Malina, 1990).

In the following decades, analyses of secular trends focused mainly on changes in aerobic performance, and they demonstrated a decline in children's and adolescents' performance in aerobic fitness tests (Cho & Kim, 2017; Prista, Marques, & Maia, 1997; Savage & Scott, 1998) as well as an increase in obesity (Booth & Lees, 2007; Fench, Story, & Jeffery, 2001). Anaerobic performance was far less widely researched (Tomkinson, Hamlin, & Olds, 2006; Tomkinson, Léger, Olds, & Cazorla, 2003). Current opinions on secular changes in children's and adolescents' fitness are highly polarized, and secular trends in children's fitness have been analyzed by fewer than three studies per year (Tomkinson, 2007). Research investigating the correlations between changes in somatic parameters, motor fitness and motor abilities (speed/agility, flexibility, strength and endurance) in specific groups (such as age groups) is even more scarce. Such studies are difficult to perform because they require a higher number of motor tests, a representative and homogeneous sample, and laborious and time‐consuming methods. The main difficulty in assessing secular trends in motor fitness is associated with the comparability of test conditions, measurement methods and equipment (Claessens & Lefevre, 1992; Malina, 1984). These problems can be partly attributed to the wide variety of the applied tests (and methodological differences even when the same tests are used) and partly to the absence of effective channels and incentives for data sharing (Tomkinson et al., 2003).

University or college students (UCS) are a specific social group, and the number of studies analyzing secular trends in UCS, particularly in first‐year students, is very limited. The first studies investigating the stature and weight of UCS were conducted between the 1950s and the 1980s (Bakwin & McLaughlin, 1964; Gyenis & Till, 1986; Malina, 1972; Polednak, 1975). Research conducted in Japan focused not only on stature and weight, but also explored parameters such as sitting height, leg and arm length, and chest girth (Ohyama et al., 1987; Takamura, Ohyama, Yamada, & Ishinishi, 1988; Yagi, Takebe, & Itoh, 1989). One of the few comprehensive studies was conducted in Belgium on first‐year students of the Catholic University of Leuven. The study involved physical education students from the academic years 1941–1942 to 1988/1989, and it evaluated 21 anthropometric variables (since 1959) and the results of 9 motor performance tests (since 1968) (Claessens & Lefevre, 1992).

Secular trends in anthropometric characteristics and motor fitness were also researched in Poland after World War II, and the relevant studies revealed a steady increase in body dimensions and motor fitness of academic youths (Milicer, Skibińska, & Skład, 1979; Piechaczek, Łaska‐Mierzejewska, & Skibińska, 1986; Pilicz, 1966, 1998). However, the changes in the somatic features and fitness levels of USC as well as the underlying causes have been rarely explored in the past two decades (Cuberek & Machova, 2009; Kaj, Tékus, Juhász, Stomp, & Wilhelm, 2015; Mleczko & Januszewski, 2009; Negasheva & Mishkova, 2005; Pribis, Burtnack, McKenzie, & Thayer, 2010). The scarcity of the relevant research is a cause for concern because selected indicators of somatic and motor development constitute the points of reference in evaluations of positive health outcomes. Academic students are an important social group, and they are worthy of a comprehensive research effort. Meanwhile, the prevalence of overweight and obesity in youths and adults has reached epidemic proportions (Delva, O‐Malley, & Johnston, 2006). Recent reports (Filkelstein, Fiebelkorn, & Wang, 2004; Hales, Carroll, Fryar, & Ogden, 2017) and studies (Flegal et al., 2010; Johnson, Dohrmann, Burt, & Mohadjer, 2014) have emphasized that the growing prevalence of overweight and obesity as well as low levels of physical activity among young people pose a serious threat to public health (Bray & Born, 2004; Delva et al., 2006; LaCaille, Dauner, Krambeer, & Pedersen, 2011).

The aim of this study was to examine changes in the anthropometric characteristics and motor fitness of first‐year university students of the University of Warmia and Mazury in Olsztyn, Poland, between 2000 and 2018. The results of the secular trend analysis are described in the article.

## 2 MATERIALS AND METHODS

### 2.1 Participants

The study was conducted in 2000–2018 on 2691 full‐time, first‐year male university students aged 19÷25 (19.98 ± 1.05 years) who were randomly selected from the population attending obligatory physical education (PE) classes at the University of Warmia and Mazury (UWM) in Olsztyn, Poland. Beginning from 2000, the study was carried out in two‐year intervals during the summer semester (April/May). Students were selected randomly on a volunteer basis, and those who wished to participate signed an informed consent form. A student who did not wish to participate in the study was replaced by another randomly drawn candidate. Only those students who were absent, for whatever reason, on the day the tests and measurements were performed, were excluded from the study. The participants were selected from among volunteers who did not take any medication or nutritional supplements, were in good health, had no history of blood diseases or diseases affecting biochemical and biomechanical factors.

### 2.2 Ethics

The research was carried out upon the prior consent of the Ethical Committee of the UWM in Olsztyn (No. 39/2011). The study involved male student volunteers who signed a written statement of informed consent.

### 2.3 Instruments and procedures

Body mass (to the nearest 0.1 kg) and body height (to the nearest 1.0 mm) were measured using a calibrated medical scale with a stadiometer (WB‐150 ZPU Tryb Wag, Poland), and the results were used to calculate the participants' BMI. Student volunteers participated in a total of 13 motor ability tests which assessed the following abilities: **speed/agility abilities**—4 × 10 m shuttle run [s], 8‐s skipping with hand clapping (SHC) test [number of claps], zig‐zag run [s]; **flexibility abilities**—standing forward bend [cm], barbell overhead trunk rotation [cm]; **strength abilities**—standing broad jump [cm], sit‐ups in 30 s [number of sit‐ups], medicine ball (4 kg) forward throw [cm], medicine ball (4 kg) backward throw [cm], pull‐ups on bar [number of pulls]; **strength endurance**—1‐min and 3‐min Burpee tests (1‐MBT, 3‐MBT) [number of cycles]; and **endurance abilities**—12‐min Cooper test on a rowing ergometer [m]. The reliability of repeated motor tests was considered high (intraclass correlation coefficient (ICC): 0.84–0.92, coefficient of variation (CV): 1.5–3.4%). These motor tests are widely used to analyze the motor abilities of different age groups, including as separate trials to assess specific motor abilities and as batteries of tests to evaluate general motor fitness (Heyward & Gibson, 2014; Podstawski, Markowski, Choszcz, Lipiński, & Borysławski, 2017; Ruiz et al., 2006; Wyss, Marti, Rossi, Kohler, & Mäder, 2007). The validity and reliability of the applied tests have been confirmed in the literature (Bianco et al., 2015; Donncha, Watson, McSweeney, & O'Donovan, 1999; Ortega et al., 2008; Pate, Burgess, Woods, Ross, & Baumgarter, 1993; Penry, Wilcox, & Yun, 2011; Podstawski et al., 2016; Podstawski et al., 2017). In each group, motor ability tests were conducted in the same order, beginning from strength, speed/agility, endurance‐strength, endurance, and ending with flexibility tests. The instructions for each test were given during the PE class, and students were allowed sufficient time to practice. The participants performed the same standard active warm‐up exercises for 10 min before tests (Frandkin, Zazryn, & Smoliga, 2010).

### 2.4 Statistical analysis

The results were processed statistically in Statistica PL v. 13 at a significance level of α = 0.05with the use of descriptive statistics (mean, SD). The following models were fit to empirical data with the least squares method:

- linear function—analyzed parameter = *a* + *b*∙*t*,
- polynomial function‐analyzed parameter = *a* + *b*∙*t* + *c*∙*t* ^2^,

The function with the highest coefficient of determination (R^2^) and statistically significant values of the y‐intercept ***a*** and coefficient of regression ***b*** was regarded as a model that best fits empirical data. The results were also expressed on a per‐year basis (Table 1) and a per‐decade basis (in the Discussion section) as means for the years of the study, to facilitate their comparison with the findings of other authors.

**TABLE 1:** Changes in the anthropometric characteristics and motor abilities of university students between 2000 and 2018

Parameters		Year										Differences		
		2000 N = 499	2002 N = 337	2004 N = 147	2006 N = 146	2008 N = 257	2010 N = 337	2012 N = 361	2014 N = 234	2016 N = 203	2018 N = 170	2000–2006	2006–2018	*D*
												Year	Year	
Anthropometric characteristics	Body mass [kg]	76.38 ± 9.36	75.14 ± 9.85	75.75 ± 10.6	74.35 ± 11.6	76.13 ± 9.55	76.97 ± 10.38	77.42 ± 10.26	77.63 ± 9.56	78.63 ± 9.36	79.72 ± 9.66	−0.46	0.45	5.37
	Body height [cm]	180.28 ± 7.08	180.36 ± 15.06	180.70 ± 5.80	181.37 ± 6.87	181.40 ± 5.93	181.45 ± 6.05	181.53 ± 6.03	181.76 ± 6.27	181.80 ± 6.77	181.99 ± 5.57	*	0.10	1.71
	BMI [kg/m^2^]	23.50 ± 4.49	23.10 ± 2.57	23.20 ± 2.78	22.60 ± 3.00	23.14 ± 2.69	23.38 ± 3.00	23.49 ± 2.80	23.50 ± 2.81	23.79 ± 3.17	24.07 ± 2.81	−0.15	0.12	1.47
Speed/agility	8‐s SHC [number of claps]	27.71 ± 3.67	28.80 ± 2.81	28.40 ± 3.55	30.80 ± 4.03	29.55 ± 2.67	29.60 ± 3.52	28.36 ± 3.56	28.34 ± 3.72	27.93 ± 4.39	27.08 ± 3.80	0.52	−0.31	−3.72
	4 × 10 m shuttle run [s]	10.76 ± 1.53	10.75 ± 0.97	10.68 ± 1.25	10.60 ± 0.94	10.78 ± 0.95	10.91 ± 0.88	11.00 ± 0.90	11.02 ± 0.91	11.05 ± 0.97	11.18 ± 1.07	−0.03	0.05	0.58
	Zig‐zag run [s]	*nr*	*nr*	25.59 ± 2.43	25.32 ± 2.62	25.52 ± 2.54	25.71 ± 2.56	25.77 ± 2.55	25.87 ± 2.63	26.22 ± 2.62	26.50 ± 2.37	−0.01	0.10	1.18
Flexibility	Downward bend [cm]	6.98 ± 5.99	7.75 ± 5.87	8.37 ± 7.27	8.52 ± 7.35	7.10 ± 5.99	6.94 ± 6.63	6.71 ± 6.73	6.53 ± 5.91	6.01 ± 6.37	5.53 ± 5.78	0.26	−0.25	−2.99
	Barbell overhead trunk rotation [cm]	*nr*	*nr*	77.71 ± 11.62	76.07 ± 13.90	78.10 ± 13.24	78.33 ± 13.39	79.08 ± 13.18	79.63 ± 13.61	80.49 ± 15.03	82.08 ± 13.77	−0.08	0.50	6.01
Strength	Standing broad jump [cm]	214.71 ± 22.69	214.02 ± 20.81	214.40 ± 21.77	215.77 ± 23.28	215.03 ± 22.64	212.00 ± 22.04	211.40 ± 22.10	213.17 ± 22.68	211.93 ± 22.94	211.40 ± 23.25	0.18	−0.36	−4.37
	30‐s sit‐ups [number of sit‐ups]	25.60 ± 4.39	26.00 ± 3.77	25.90 ± 4.59	27.30 ± 3.40	26.21 ± 4.42	25.52 ± 4.27	25.45 ± 4.36	24.93 ± 4.70	24.54 ± 4.58	23.90 ± 4.09	0.28	−0.28	−3.4
	Medicine ball backward throw [cm]	1029.0 ± 200.61	1043.8 ± 200.92	1070.8 ± 192.75	1083.0 ± 187.56	1049.0 ± 207.19	1041.4 ± 190.08	1025.3 ± 195.03	1013.0 ± 180.72	1006.6 ± 174.75	1001.8 ± 194.85	9.00	−6.77	−81.2
	Medicine ball forward throw [cm]	*nr*	*nr*	899.59 ± 165.33	953.20 ± 164.08	910.81 ± 166.41	882.75 ± 166.87	872.56 ± 173.39	857.09 ± 161.23	856.50 ± 166.93	837.43 ± 154.69	2.68	−9.65	−115.77
	Pull‐ups [number of pull‐ups]	4.86 ± 4.02	5.05 ± 3.62	5.06 ± 4.18	5.73 ± 4.08	5.44 ± 3.71	5.06 ± 3.86	5.16 ± 3.94	4.80 ± 3.64	4.72 ± 3.65	4.41 ± 3.76	0.15	−0.11	−1.32
Strength endurance	1‐MBT [number of cycles]	25.88 ± 4.16	25.49 ± 3.08	26.86 ± 4.21	27.90 ± 4.59	27.49 ± 3.25	26.55 ± 4.31	25.98 ± 4.37	25.90 ± 4.89	25.16 ± 4.89	25.17 ± 4.19	0.34	−0.23	−2.73
	3‐MBT [number of cycles]	*nr*	*nr*	58.25 ± 8.07	59.62 ± 9.22	59.46 ± 8.33	59.03 ± 8.87	58.90 ± 8.93	58.90 ± 9.66	57.17 ± 9.58	56.68 ± 8.92	0.07	−0.25	−2.94
Endurance	12‐min rowing ergometer test [m]	*nr*	*nr*	2637.84 ± 273.92	2687.00 ± 343.28	2601.90 ± 322.06	2601.40 ± 300.27	2593.50 ± 308.22	2586.12 ± 298.80	2562.90 ± 293.61	2509.00 ± 327.48	2.46	−14.83	−128.84

***Note*:***nr*, trials were not conducted in a given academic year; *, difference between 2000 and 2018; *D*, difference between the minimum and maximum values.

## 3 RESULTS

**FIGURE 1:**
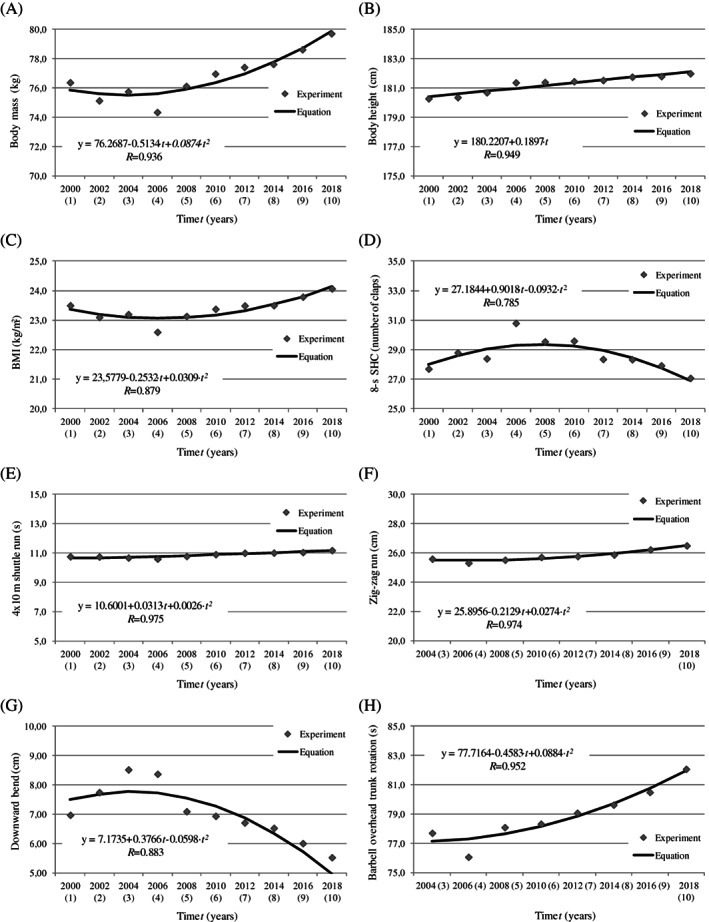
Secular trends in anthropometric characteristics, speed/agility and flexibility abilities between 2000 and 2018

**FIGURE 2:**
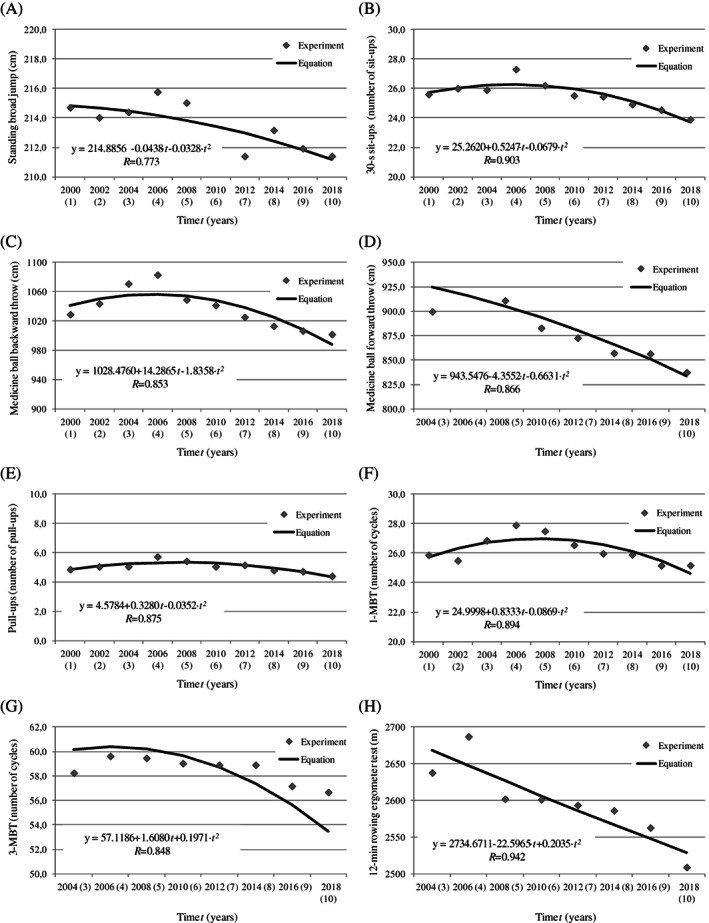
Secular trends in strength, strength endurance and endurance abilities between 2000 and 2018

The average values of anthropometric characteristics and motor abilities in each year of the study and the average changes in the examined variables (per year) are presented in Table 1. The results were analyzed in two time periods (2000–2006 and 2006–2018) because the highest scores in all motor tests were noted in 2006. Therefore, 2006 was regarded as a critical point—a steady improvement in results was observed between 2000 and 2006, after which motor fitness scores continued to decrease until 2018. The changes noted between 2000 and 2018 and the accompanying secular trend equations are presented graphically in Figures 1 and 2.

### 3.1 Analysis 1: Changes in anthropometric characteristics

Body mass values were lowest in 2006 (74.4 kg), after which they increased steadily to reach 79.7 kg in 2018. The difference between the minimum and maximum body mass values was determined at 5.4 kg. On average, body mass decreased by 0.5 kg/year (0.5%) between 2000 and 2006, and it increased by 0.5 kg/year (0.6%) between 2006 and 2018. Similar trends were noted in BMI values which decreased by 0.2 kg/m^2^/year (0.7%) between 2000 and 2006, and increased by 0.1 kg/m^2^/year (0.5%) between 2006 and 2018 on average. BMI values were within the norm in all years of the study, and the difference between the minimum and maximum values was determined at 1.5 kg/m^2^. Changes in body height followed a different trend than the remaining anthropometric characteristics. Beginning from 2000, body height increased steadily by 0.1 cm/year (0.1%) and the difference between the minimum and maximum values was determined at 1.7 cm (Table 1).

### 3.2 Analysis 2: Changes in speed/agility abilities

Between 2000 and 2006, the results scored by university students in speed/agility tests (excluding the zig‐zag run) improved by 0.52 claps/year (1.7%) in the 8‐s—SHC test, and by −0.03 s/year (0.3%) in the 4x10 m shuttle run on average. Between 2006 and 2018, the results scored by the evaluated subjects in the 8‐s—SHC, 4x10 m shuttle run and the zig‐zag run decreased by −0.31 claps (1.1%), 0.05 s (0.4%) and 0.10 s (0.4%) per year, respectively. The corresponding differences between the minimum and maximum values were determined at −3.7 claps, 0.58 s and 1.18 s.

### 3.3 Analysis 3: Changes in flexibility abilities

Between 2000 and 2006, the results of the downward bend test improved steadily by 0.26 cm/year (2.8%) on average. Between 2006 and 2018, the results of the downward bend test and the barbell overhead trunk rotation test declined by 0.25 cm (4.3%) and 0.50 cm (0.6%) per year, respectively. The differences between the minimum and maximum values were determined at 2.99 and 6.01 cm, respectively.

### 3.4 Analysis 4: Changes in strength abilities

The scores noted in strength ability tests improved gradually between 2000 and 2006 by 0.18 cm/year (0.1%) in the standing broad jump, by 0.28 sit‐ups/year (1.0%) in 30‐s sit‐ups, by 9.0 cm/year (0.8%) in the medicine ball backward throw, and by 0.15 pull‐ups/year (2.5%) in the pull‐ups on bar test. Between 2006 and 2018, the respondents' performance in all strength ability tests deteriorated by 0.36 cm/year (0.2%) in the standing broad jump, by 0.28 sit‐ups/year (1.2%) in 30‐s sit‐ups, by 6.77 cm/year (0.7%) in the medicine ball backward throw, by 9.65 cm/year (1.2%) in the medicine ball forward throw, and by 0.11 pull‐ups/year (2.9%) in the pull‐ups on bar test. The differences between the minimum and maximum values in the above tests reached 4.37 cm, 3.4 sit‐ups, 81.2 cm, 115.77 cm, and 1.32 pull‐ups, respectively.

### 3.5 Analysis 5: Changes in strength endurance and endurance abilities

Between 2000 and 2006, the tested students improved their performance in the 1‐MBT by 0.34 cycles/year (1.2%). A steady decrease in the results of all strength endurance and endurance tests was noted between 2006 and 2018 when the analyzed values decreased by 0.23 cycles/year (0.9%) in 1‐MBT, by 0.25 cycles/year (0.4%) in 3‐MBT, and by 14.83 m/year (0.6%) in the 12‐min rowing ergometer test. The differences between the lowest and the highest scores reached 2.7 cycles, 2.94 cycles, and 128.84 m, respectively.

### 3.6 Analysis 6: Secular trends for anthropometric characteristics—regression equations

Changes in body mass and BMI values between 2000 and 2018 were described by a parabola. The regression equations for body mass and BMI clearly indicate that both parameters decreased between 2000 and 2006 and increased between 2006 and 2018. Changes in body height had a linear character, and a gradual increase in this parameter produced a significant positive secular trend (Figure 1).

### 3.7 Analysis 7: Secular trends for motor characteristics—regression equations

Second‐order parabolic equations were developed for the values measured between 2000 and 2018, including the results of all speed/agility tests (8‐s—SHC, 4 x 10 m shuttle run, zig‐zag run), flexibility tests (downward bend, barbell overhead trunk rotation), strength tests (standing broad jump, 30‐s sit‐ups, medicine ball backward and forward throws, pull‐ups), as well as strength endurance and endurance tests (1‐MBT, 3‐MBT, 12‐min rowing ergometer test). All foci of the parabola representing the best results of motor tests were noted in 2006 (Figures 1 and 2).

### 3.8 Analysis 8: Secular changes in the relationships between anthropometric and motor characteristics

An analysis of the results noted in all years of the study revealed significant indirect correlations between motor abilities and body mass, where the correlation coefficient (*r*) ranged from −0.98 to 0.99, and between motor abilities and BMI, where the value of *r* ranged from −0.98 to 0.98 (both at *P* < 0.05). A positive correlation denotes an increase in test scores expressed in terms of time (4x10 m shuttle run and zig‐zag run test) and distance (barbell overhead trunk rotation test), and it points to a gradual decline in the speed/agility and flexibility abilities, respectively, of the tested students.

## 4 DISCUSSION

The aim of this study was to examine the anthropometric characteristics and motor abilities of Polish university students and to determine changes in the studied parameters between 2000 and 2018. Anthropometric characteristics such as body mass and BMI in young men decreased between 2000 and 2006, whereas a steady increase in these traits was noted between 2006 and 2018. Body height continued to increase in all years of the study (2000–2018).

The current (2018) generation of first‐year students at the UWM is significantly taller and heavier than the generation tested 18 years ago (2000). During the 18‐year study, body height increased by 1.7 cm on average, which translates into an estimated rate of secular increase of 0.1 cm per year and 1.0 cm per decade. In general, the increase in stature is smaller than that reported in university students between 1945 and the early 1990s (Bakwin & McLaughlin, 1964; Claessens & Lefevre, 1992; Gyenis & Till, 1986; Malina, 1990; Polednak, 1975; Takamura et al., 1988; Yagi et al., 1989). A comparison of our results with previous findings revealed a continued increase in the body height of first‐year students in each decade, but the rate of the observed growth was lower than in the preceding years. These findings are consistent with the observations made by Polish anthropologists who predicted that the trend of increasing height would gradually decline and that body mass and average BMI values would continue to grow in the Polish population (Szklarska, Kozieł, Bielicki, & Welon, 2004). The predictions concerning changes in body mass and BMI values (Szklarska et al., 2004) are also validated by the results of the present study. Body mass and BMI continued to decrease between 2000 and 2006, whereas a steady increase of less than 0.5 kg/year and 4.5 kg/decade was noted between 2006 and 2018. The observed increase in the body mass of UWM students was greater than that noted in earlier research (Bakwin & McLaughlin, 1964; Claessens & Lefevre, 1992; Gyenis & Till, 1986; Malina, 1990; Polednak, 1975; Yagi et al., 1989). A study of Russian university students also demonstrated a greater increase in body mass in recent years (Negasheva & Mishkova, 2005). According to Negasheva and Mishkova (2005), the increase in the anthropometric characteristics (body mass and BMI) of contemporary young men can be partly explained by the presence of overweight students in the sample or by the influence of social selection because students from families with a high social status were heavier than their peers. The results of the current study suggest that a significant increase in body mass and body height is the main secular trend in first‐year university students. Similar observations were made in other studies of university students (Kaj et al., 2015; Mleczko & Januszewski, 2009; Negasheva & Mishkova, 2005) as well as children and adolescents (Albon, Hamlin, & Ross, 2010; Matton et al., 2007).

The present findings suggest that the motor fitness of young men has been deteriorating, which, according to Negasheva and Mishkova (2005), can be attributed to the loss of muscle tissue and replacement of muscle tissue by adipose tissue. However, the above observations could not be confirmed because this study did not involve a body composition analysis (determination of the percentage of fat and muscle tissue) or skeleton measurements as a basis for evaluating the participants' physical development (Greil & Lange, 2007). An analysis of BMI values points to clear change trends in the ratio of body mass and body height which is a reliable indicator of changes in the average weight and height of a population (Romero‐Corral et al., 2008). The steady increase in the BMI values of young males gives cause for concern, and it is probably associated with the specific lifestyle of university students which is characterized by low levels of physical activity and high food intake. First‐year university students are particularly likely to lead a sedentary lifestyle and consume excess calories (Bray & Born, 2004; LaCaille et al., 2011; Vella‐Zarb & Elgar, 2009). It should also be noted that BMI is strongly determined by absolute body mass which is governed by physiological regulatory mechanisms. Body mass is less genetically conditioned than body height, which is why it changes more rapidly in response to changes in lifestyle, physical activity, nutrition or disease, whereas changes in body height under the influence of environmental factors proceed more gradually and are irreversible (Piontek, 2004). The results of the present study provide clear evidence for these correlations: body mass increased by 5.61% per decade, whereas the corresponding increase in body height was determined at only 0.52%.

An analysis of the relationships between anthropometric characteristics and motor abilities revealed that the evaluated parameters were most satisfactory in 2006, when students were characterized by the lowest body mass (74.35) and lowest BMI (22.6 kg/m^2^). The above traits were least satisfactory in 2018. An analysis of combined data for all years of the study demonstrated significant indirect correlations between motor abilities vs body mass and BMI. Similar correlations between BMI and VO_2max_ values were reported in American college students by Pribis et al. (2010). Numerous studies have shown that high body mass exerts a negative impact on the motor fitness test scores of female and male university students (Mohammadi & Saberi, 2016; Podstawski, Markowski, Choszcz, & Żurek, 2016). Special attention should be paid to a study by Podstawski, Markowski, and Clark (2020) who analyzed the correlations between selected anthropometric features (body mass, body height, BMI) and motor abilities in a group of first‐year university students with nearly identical characteristics. In their study, body mass and BMI were significantly negatively correlated with the results of most motor tests. However, Borysławski, Podstawski, Ihasz, and Żurek (2020) demonstrated that when physical activity levels were included in the analysis, this parameter was the key determinant of endurance‐strength abilities.

Beginning from 2006, a steady decline in all of the evaluated motor abilities (speed/agility, flexibility, strength, strength endurance and endurance) was observed in 12 successive years. The greatest decrease was noted in sagittal spinal flexibility (4.24% per year and 42.8% per decade in the downward bend test), followed by strength abilities (2.49% per year and 24.94% per decade in the pull‐ups on bar test), speed abilities (1.14% per year and 11.45% per decade in the 8‐s SHC test) and strength endurance abilities (0.9% per year and 9.04% per decade in 1‐MBT). The smallest decline was noted in the results of the test measuring the explosive strength of lower limbs (0.17% per year and 1.72% per decade in the standing broad jump). A continued decrease in the physical strength abilities (grip strength in kg measured in the hand dynamometry test) of Russian male university students was also reported in 1960–69, 1991 and 2003–2003 (Negasheva & Mishkova, 2005). Strength abilities are a special group of motor abilities because a decrease in muscle mass and physical strength leads to a general decline in motor development and, consequently, loss of adaptability to environmental conditions.

Yagi et al. (1989) analyzed secular changes in the motor abilities of Japanese students of Kyoto University in Japan between 1964 and 1987. They noted a positive trend in vital capacity, standing long jump and the 100 m dash. A significant decrease (negative trend) was observed in left and right handgrip strength, whereas no differences were reported in back muscle strength, ball throwing skills and push‐ups. A comprehensive study of first‐year students of the Catholic University of Leuven from the academic years 1941–1942 to 1988/1989 was conducted in Belgium. The study evaluated 21 anthropometric variables (since 1959) and the results of 9 motor performance tests (since 1968). Students from the most recent cohort were characterized by significantly narrower shoulders, smaller hip circumference and thicker triceps skinfolds than those evaluated 25 years earlier. The investigated subjects scored higher in flexibility texts, several strength tests and running speed tests than those examined 15 years earlier. However no changes were observed in the average limb movement speed (Claessens & Lefevre, 1992).

Observations of Hungarian university students conducted over a period of 15 years (1998–2012) also revealed a decline in the results of the Flamingo test, plate tapping test, sit and reach test, and sit‐up test. However, an improvement was noted in the results of the handgrip strength test and the bent arm hanging test (Kaj et al., 2015). The performance of Slovakian male university students in the dash test varied between 1991 and 2006, and the overall trend was neither positive nor negative during the 15‐year study (Cuberek & Machova, 2009). Similar fluctuations in selected motor abilities were observed in the current study, but in two‐year intervals. The motor abilities of university students are sensitive to environmental stimuli and are thus more labile. Motor abilities can both increase and decrease subject to short‐term changes in physical activity levels.

The superiority of the results noted in 2006, including the body mass‐body height ratio (lowest body mass of 74.35 kg and lowest BMI of 22.6 kg/m^2^ which were located in the middle of the normal range of values) and the results of motor tests, over the remaining years of the study is quite unusual, and it could be largely attributed to socioeconomic factors. In 2006, Polish citizens enjoyed much higher socioeconomic status than in the preceding and successive years. In the UWM in Olsztyn, 2006 was a year with the highest number of students in the university's history, the highest number of sports events, sports clubs and athletic scholarships. The students who participated in the Polish Academic Championships were entitled to free accommodation in academic dormitories and free meals in the university canteen. Between 2000 and 2006, most faculties organized 60 up to 120 hours of obligatory physical education classes, but the number of class hours decreased significantly in the following years. The Department of Physical Education and Sport also organized the highest number of free facultative sports activities in 2006. In 2018, the physical education curriculum was reduced to 30 hours during the entire study program. Some classes consisted of lectures, and physical education was completely eliminated from the curricula of some faculties, including the Faculty of Medicine (Podstawski, 2018). Therefore, the programs and facilities available to students in 2006 were more conducive to physical training and sports promotion than in the remaining years of the study.

## 5 STRENGTHS AND LIMITATIONS

The variations in the somatic and motor development of a population can be regarded as biological indicators of adaptability to environmental conditions. The results of this study provide valuable and reliable data that complement previous cross‐sectional studies into the somatic and motor development of Polish university students, which constituted the only large‐scale studies investigating the anthropometric characteristics and motor abilities of Polish university students between 2000 and 2018. The main strength of this study is that it was conducted in two‐year intervals over a period of 18 years. The main limitation is the lack of a body composition analysis which could not be performed in the initial years of the study (before 2008) when a body composition analyzer based on bioelectrical impedance was not available. Due the repeated measurements of body mass and height, as well as a relatively high number of motor tests, additional anthropometric measurements involving older methods (such as skinfold tests) would be very difficult to perform in a reliable manner within a relatively short time.

## 6 CONCLUSIONS

The regression analysis confirmed the linear character of changes in university students' body height in the evaluated period (2000–2018). The changes in the remaining anthropometric characteristics (body mass, BMI) and motor abilities of university students can be described with a quadratic equation. Secular changes in body height were noted in male students who participated in the study between 2000 and 2018. Body mass and BMI values decreased between 2000 and 2006, whereas a steady increase in these parameters was noted between 2006 and 2018. The changes in all motor abilities (speed/agility, flexibility, strength, strength endurance and endurance abilities) followed an opposite trend to the changes in body mass and BMI, that is, an improvement was noted between 2000 and 2006, whereas a decline was observed between 2006 and 2018. The trend of increasing body height has been maintained in first‐year university students in each decade, but unlike body mass and BMI, the rate of increase in body height was lower than in the preceding years. The changes noted in the experimental period should also be taken into account when analyzing stratified trends in selected socioeconomic factors.

## AUTHOR CONTRIBUTIONS

**Robert Podstawski:** Conceptualization; data curation; formal analysis; funding acquisition; investigation; methodology; software; supervision; validation; writing‐original draft; writing‐review and editing. **Piotr Żurek:** Data curation; funding acquisition; investigation; writing‐review and editing.

## CONFLICT OF INTEREST

The authors declare that they have no conflict of interest.
